# CAR-NK cell-derived exosomes in cancer therapy: bridging cellular immunotherapy and nanomedicine

**DOI:** 10.3389/fimmu.2026.1866170

**Published:** 2026-10-27

**Authors:** Mazdak Ganjalikhani Hakemi, Zohreh Saltanatpori, Lingqi Zhou, Xiangbo Zeng, Lu Sun, Ziwei Liao

**Affiliations:** 1 Regenerative and Restorative Medicine Research Center (REMER), Research Institute for Health Sciences and Technologies (SABITA), Istanbul Medipol University, Istanbul, Türkiye; 2 Translational Ophthalmology Research Center, Farabi Eye Hospital, Tehran University of Medical Sciences, Tehran, Iran; 3 Heart Center and Institute of Pediatrics, Guangzhou Medical University Guangzhou Women and Children’s Medical Center, Guangzhou, China; 4 Department of Hematology, National Key Clinical Specialty, Guangzhou First People’s Hospital, Institute of Blood Transfusion and Hematology, Guangzhou Medical University, Guangzhou, China; 5 Department of Hematology/Oncology, Guangzhou Medical University Guangzhou Women and Children’s Medical Center, Guangzhou, China

**Keywords:** cancer immunotherapy (CIT), CAR-NK cell, CAR-NK-derived EVs, exosome, nanomedicine

## Abstract

Chimeric antigen receptor-engineered natural killer (CAR-NK) cell therapy has emerged as a promising cancer treatment by combining the antigen specificity of CAR engineering with the intrinsic cytotoxicity of NK cells. Compared with CAR-T cell therapy, CAR-NK cells exhibit a lower risk of graft-versus-host disease (GVHD), cytokine release syndrome (CRS), and immune-related toxicities while offering the potential for allogeneic “off-the-shelf” applications. Nevertheless, their therapeutic efficacy remains limited by insufficient persistence, poor trafficking, and the immunosuppressive tumor microenvironment (TME). Extracellular vesicles (EVs), particularly small EVs (sEVs) derived from CAR-NK cells may retain tumor-targeting features and cytotoxic cargo of their parental cells while offering potential advantages of a cell-free format, including reduced proliferative risk, improved tissue penetration, enhanced biological stability, and the capacity to deliver therapeutic proteins, nucleic acids, and small molecules. Emerging preclinical evidence demonstrates that CAR-NK-derived sEVs can directly eliminate tumor cells while simultaneously modulating the TME, making them an attractive cell-free immunotherapeutic platform; however, their safety and pharmacological properties require dedicated validation. Despite their considerable promise, several challenges remain, including scalable manufacturing, standardized characterization, vesicle heterogeneity, cargo loading efficiency, biodistribution, and pharmacokinetic optimization. This review summarizes recent advances in CAR-NK-derived sEV research, critically discusses their biological properties, engineering strategies, preclinical evidence, translational challenges, and future clinical prospects, highlighting their emerging role at the intersection of cancer immunotherapy and nanomedicine.

## 1 Introduction

Cancer remains a global health challenge, with high morbidity and mortality rates, emphasizing the need for innovative therapeutic strategies. Although conventional treatments such as chemotherapy and radiotherapy prolong survival, their nonspecific nature often results in significant off-target effects and resistance (1, 2).

Chimeric antigen receptor (CARs) are synthetic receptors which can direct T cells to target cells expressing specific antigens (3). Today, CAR-T has become a pivotal advancement in immunotherapy, particularly with remarkable success in treating hematologic malignancies (3–5).

Despite all advancements, CAR-T cells face limitations, including severe toxicity, complex manufacturing, and reduced efficacy against solid tumors (6, 7). Moreover, CAR-Ts are associated with significant side effects such as cytokine release syndrome (CRS) and graft-versus-host disease (GVHD).

Natural killer (NK) cells with intrinsic antitumor activity, do not require antigen presentation via the major histocompatibility complex (MHC), and has a lower risk of GVHD in allogeneic transplantation due to limited clonal expansion. So that CAR-engineered NK (CAR-NK) cells can offer several advantages over CAR-T cells. In addition, CAR-NK cells are able to modulate the tumor microenvironment (TME) and penetrate solid tumor effectively, highlighting their potential as a promising approaches for treatment of advanced and malignant cancers (3). Although CAR-NK shows great potential, it still faces several challenges, particularly in treating solid tumors. Major challenges are optimizing CAR constructs for NK-specific signaling, enhancing persistence and activation, and overcoming the immunosuppressive TME (8, 9).

On the other hand, advances in nanomedicine have accelerated the development of extracellular vesicle (EV)-based therapeutics. According to the Minimal Information for Studies of Extracellular Vesicles (MISEV2023) guidelines, the generic term “extracellular vesicles (EVs)” is recommended unless a specific biogenesis pathway has been unequivocally demonstrated. Accordingly, throughout this review, the term “small extracellular vesicles (sEVs)” is preferentially used when discussing general concepts, whereas “exosomes” is retained when referring to studies in which the investigated vesicles were explicitly described as exosomes by the original authors. EVs derived from CAR-T (CAR-T-EVs) or CAR-NK cells (CAR-NK-EVs) integrate the targeting specificity of engineered immune cells with the favorable delivery properties and biocompatibility of nanoscale vesicles, thereby offering an encouraging cell-free strategy for cancer immunotherapy. Compared with living cell therapies, CAR-NK-derived sEVs are not susceptible to phenotypic exhaustion, uncontrolled proliferation, or tumor-mediated functional reprogramming after administration, while retaining many of the antitumor properties of their parental cells (9–14).

CAR-NK cell-derived EVs may retain aspects of the tumor-killing activity of their parental NK cells and can potentially be further modified using physical, chemical, or biological approaches to enhance their therapeutic properties (11, 13).

Compared with viable cell therapies, EVs may offer advantages related to their non-proliferative nature, storage and formulation; however, their safety, scalability, and therapeutic consistency must be established independently through rigorous product-specific studies (12).

This review critically summarizes current advances in CAR-NK-derived extracellular vesicles, with particular emphasis on their biological characteristics, engineering strategies, mechanisms of antitumor activity, translational challenges, and future opportunities. By integrating concepts from cellular immunotherapy, extracellular vesicle biology, and nanomedicine, we aim to highlight both the promise and the remaining barriers to clinical translation of this emerging therapeutic platform.

## 2 Overview of CAR-NK cell-derived extracellular vesicles

### 2.1 Biology of CAR-NK cells

NK cells are key effectors of innate immunity that recognize and eliminate transformed or infected cells without prior antigen sensitization or major histocompatibility complex (MHC) restriction. Their activation is governed by the balance between activating receptors - including NKG2D, NKp30, NKp44, NKp46, DNAM-1 (CD226), and CD16 (FcγRIII) - and inhibitory receptors such as killer cell immunoglobulin-like receptors (KIRs) and CD94/NKG2A, which recognize MHC class I molecules expressed on healthy cells. Tumor cells frequently downregulate MHC-I while upregulating stress-induced ligands, thereby shifting this balance toward NK-cell activation and triggering rapid cytotoxic responses through perforin/granzyme release, death receptor signaling, and cytokine secretion, including IFN-γ and TNF-α (7, 10, 14).

Unlike T lymphocytes, whose activation requires antigen processing and peptide presentation by MHC molecules together with co-stimulatory signals, NK cells can rapidly recognize and eliminate abnormal cells through receptor-mediated sensing of cellular stress. Dendritic cells, by contrast, depend on an even more elaborate, multistep sequence of antigen uptake, intracellular processing, and cross-presentation via MHC class I/II to prime adaptive T-cell responses, a pathway that affords tumors additional opportunities for immune evasion; this comparative complexity further underscores the speed and simplicity of NK-cell-mediated recognition as a rationale for prioritizing NK cells as both the CAR-engineering platform and the EV source. This MHC-independent surveillance complements CAR-mediated antigen recognition rather than replacing the endogenous cytotoxic machinery of NK cells. Consequently, CAR engineering provides NK cells with dual antitumor functionality by integrating antigen-specific targeting with their intrinsic ability to respond to tumor immune evasion, making them an attractive platform for both adoptive cellular therapy and the generation of therapeutic extracellular vesicles (9, 14–17).

Accordingly, CAR-NK-derived extracellular vesicles inherit many membrane-associated receptors and cytotoxic molecules from their parental cells while eliminating several limitations associated with living-cell therapies. Compared with T-cell-derived extracellular vesicles, NK-cell-derived extracellular vesicles are naturally enriched in perforin, granzymes, granulysin, and death receptor ligands, further supporting their development as highly effective cell-free immunotherapeutics (12, 18–20).

The choice of CAR-NK cells as the parental source may provide additional biological advantages over CAR-T cells for EV-based immunotherapy. CAR-NK cell-derived EVs combine CAR-mediated antigen recognition with the cytotoxic and immunomodulatory cargo characteristic of NK cells, including perforin, granzymes, granulysin, and death receptor ligands, while avoiding the need to administer viable effector cells. In contrast, CAR-T-derived EVs may reflect the distinct molecular and functional properties of T-cell-derived vesicles. However, these differences currently provide a biological rationale rather than evidence of definitive superiority, as direct head-to-head comparisons of CAR-NK-derived and CAR-T-derived EVs remain limited (18–21).

CAR-NK cells integrate three key components: an extracellular single-chain variable fragment (scFv) for antigen binding, a spacer domain for optimal receptor orientation, and an intracellular activation domain to trigger cytotoxicity. This design enables precise targeting of TAAs while leveraging the innate tumor-killing mechanisms of NK cells (9, 22). These features enable CAR-NK cells to target tumors with high precision, reducing off-target effects compared with traditional NK cell therapies.

One of the principal advantages of CAR-NK cells over CAR-T cells is their favorable safety profile, characterized by a lower incidence of cytokine release syndrome (CRS), graft-versus-host disease (GVHD), and other immune-related toxicities. In addition, CAR-NK cells can be generated from healthy donors for allogeneic “off-the-shelf” use, facilitating broader clinical applicability (17, 23–25). These properties concern viable CAR-NK-cell therapy and should not be automatically transferred to CAR-NK cell-derived EV products.

Furthermore, CAR-NK cells offer increased ability to infiltrate solid tumors. This is particularly important given that solid tumors often present a challenging environment for immune cells due to the presence of immunosuppressive factors, such as hypoxia and nutrient deprivation, within the TME (9). Studies have shown that CAR-NK cells can not only directly target and kill tumor cells but also modulate the TME, making the environment more conducive to immune cell activity and improving the overall antitumor response (26, 27).

In addition to their direct tumor-killing activity, CAR-NK cells can enhance the function of other immune cells in the TME, including T cells and dendritic cells. This dual mechanism—direct tumor cytotoxicity coupled with immune modulation—further strengthens their potential as a powerful tool in solid tumor therapy (3, 16). As research into CAR-NK cell biology advances, understanding how to optimize their expansion, persistence, and ability to function within the suppressive TME will be crucial for maximizing their therapeutic potential.

### 2.2 Extracellular vesicles: biogenesis and characteristics

Exosomes are small extracellular vesicles (EVs), typically ranging from 30 to 150 nanometers in diameter that play crucial roles in cellular communication. They are secreted by a multitude of cell types, including immune cells such as NK cells, and are increasingly recognized for their importance in both physiological and pathological processes (19, 28). The biogenesis of EVs begins within the endosomal pathway, specifically in the formation of multivesicular bodies (MVBs). These MVBs encompass intraluminal vesicles (ILVs) that bud inward from the membrane of the early endosome. When MVBs mature, they are transported to the plasma membrane of the cell, where they undergo fusion with the lipid bilayer, resulting in the release of EVs into the extracellular space (29, 30).

Isolation and characterization of therapeutic EVs require standardized, orthogonal approaches to ensure reproducibility and product quality. Common isolation strategies include differential ultracentrifugation, size-exclusion chromatography, density-gradient separation, and increasingly scalable tangential-flow filtration or related downstream technologies. Characterization should combine particle concentration and size distribution measurements, such as nanoparticle tracking analysis, with morphological assessment and protein profiling. For CAR-NK cell-derived EVs, characterization may additionally include multiple EV-associated markers, such as CD63, CD81, CD9, TSG101 and ALIX, together with assessment of relevant NK-cell-associated proteins and cellular contaminants. Importantly, canonical EV markers alone are insufficient to establish EV identity or purity because these proteins are not uniquely restricted to a single EV subtype; therefore, complementary assessment of particle properties, morphology, protein composition, and contaminating cellular components should be performed in accordance with MISEV2023 recommendations (31–33).

EVs serve as pivotal vehicles for intercellular communication, enabling the transfer of a range of biomolecules, such as proteins, lipids, and nucleic acids, including messenger RNA (mRNA) and microRNA (miRNA) (34–36). This unique composition is reliable indicators of cellular function and health (37). By delivering bioactive signals, EVs can modulate many key biological processes including immune responses, tumor progression, and inflammation (38). These vehicles by delivering signals can also effect on target cell behavior, promoting proliferation, migration, or apoptosis, highlighting their essential role in both normal and pathologic states (39, 40).

Beyond serving as intercellular messengers, extracellular vesicles have emerged as attractive cell-free therapeutic platforms because they can retain many biological functions of their parental cells while avoiding several limitations associated with living-cell therapies. Unlike immune cells, EVs are non-proliferative and, because they are cell-free, are not expected to undergo the phenotypic exhaustion or tumor-mediated functional reprogramming that can affect adoptively transferred immune cells within the tumor immune microenvironment (TIME). This distinction is clinically relevant: cell-based vaccines, such as dendritic cell (DC) vaccines, can traffic into the TME following administration, where local immunosuppressive cues can reprogram them toward a tolerogenic or pro-tumor phenotype that favors tumor growth; EVs, as non-living particles, cannot proliferate or undergo the same type of cellular phenotypic reprogramming (41). In addition, their nanoscale size facilitates diffusion through dense extracellular matrices and enables efficient delivery of cytotoxic proteins and regulatory RNAs directly to tumor and stromal cells, contributing to immune activation and remodeling of the immunosuppressive TME (12, 19, 42, 43).

Furthermore, their nanoscale size may enable them to cross biological barriers, including the blood-brain barrier (BBB), and may penetrate the more permissive blood-tumor barrier (BTB); however, the extent of BTB penetration and its relationship to therapeutic efficacy remain dependent on EV properties, tumor model, administration route, and experimental methodology (29, 42, 44). As research continues to elucidate their diverse biological functions, engineered CAR-NK cell-derived EVs are increasingly recognized as attractive next-generation immunotherapeutic nanocarriers (**Figure 1**). The structural composition, representative cargo, and major functional properties of CAR-NK-derived EVs are summarized in **Figure 2**.

**Figure 1:**
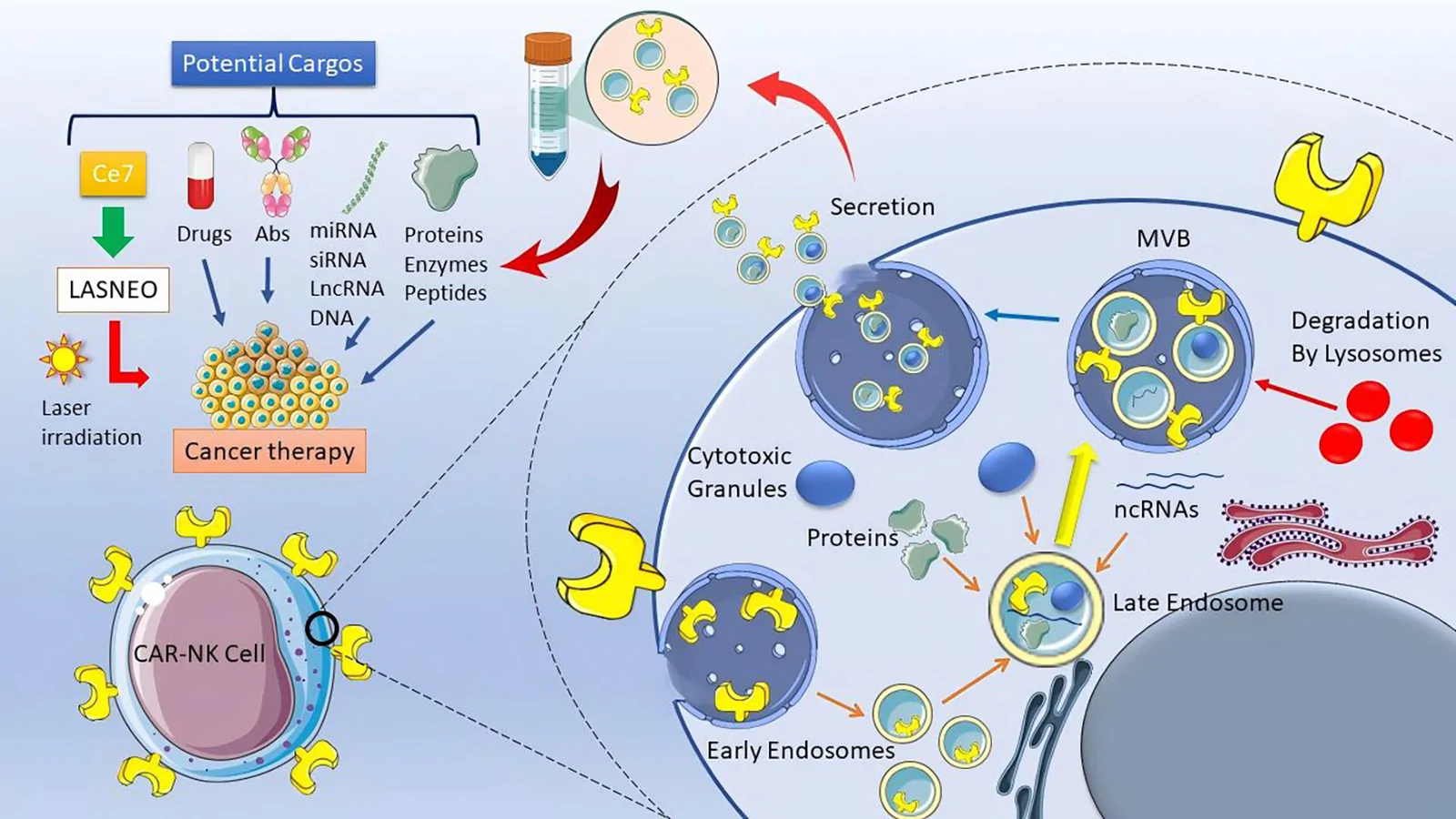
This figure illustrates the biogenesis process and the characteristics of CAR-NK cell-derived EVs. The stages of EV biogenesis within a CAR-NK cell are depicted, including early endosomes, late endosomes, and multivesicular bodies (MVBs). The figure also highlights potential therapeutic cargos, including anticancer drugs, monoclonal antibodies, miRNAs, siRNAs, lncRNAs, DNA, proteins, enzymes, small peptides, and photosensitizers. CAR-NK cell-derived EVs, CAR-NK Cell-Derived Exosomes; Exos, Exosome; MVBs, multivesicular bodies; mAbs, monoclonal antibodies; lncRNAs, long noncoding RNAs; LASNEO, Light-Activatable Silencing NK-Derived Exosome.

**Figure 2:**
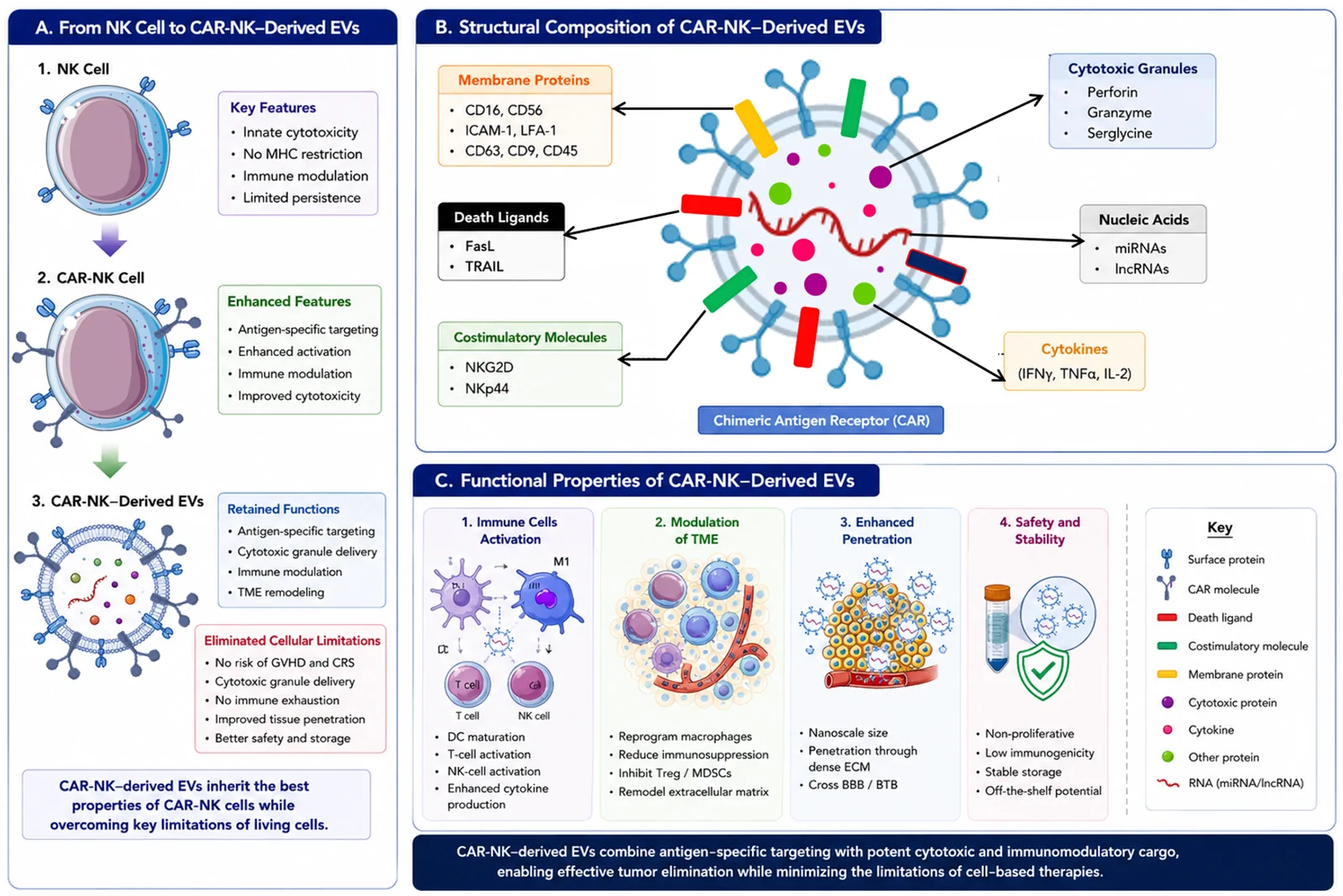
Structural composition and functional properties of CAR-NK-derived extracellular vesicles (EVs). **(A)** Generation of CAR-NK cell-derived EVs from parental NK cells following CAR engineering, illustrating the retention of antitumor functions while overcoming key limitations of living-cell therapies. **(B)** Schematic representation of the major membrane-associated and intravesicular components of CAR-NK cell-derived EVs, including CAR molecules, membrane proteins, cytotoxic proteins, nucleic acids, cytokines, and immunomodulatory molecules. **(C)** Principal proposed functions of CAR-NK cell-derived EVs, including immune activation, modulation of the tumor microenvironment, enhanced tumor penetration, and the potential advantages and limitations of a cell-free immunotherapeutic format.

### 2.3 CAR-NK-derived extracellular vesicles and their advantages

Clinical experience with CAR-NK cells provides important context for the parental cellular platform but should not be interpreted as clinical evidence for CAR-NK cell-derived EVs. In the pivotal cord-blood-derived CD19-directed CAR-NK study, 8 of 11 treated patients (73%) achieved an objective response and 7 of 11 (64%) achieved a complete response, with no reported cytokine release syndrome, neurotoxicity, or GVHD (45). Other early CAR-NK studies have reported activity in hematological malignancies, whereas clinical experience in solid tumors remains limited (21, 46).

Despite these encouraging clinical outcomes, CAR-NK cell therapy continues to face important challenges, including limited *in vivo* persistence, insufficient expansion after infusion, manufacturing scalability, and reduced efficacy within the immunosuppressive tumor microenvironment of solid tumors (8, 9). These limitations have stimulated growing interest in CAR-NK-derived extracellular vesicles as a complementary cell-free therapeutic strategy capable of preserving many of the antitumor properties of CAR-NK cells while potentially overcoming several obstacles associated with living-cell therapies (12, 42, 46).

Engineering CAR-NK-derived extracellular vesicles begins with the genetic modification of NK cells to express CAR constructs directed against tumor-associated antigens (TAAs). During vesicle biogenesis, these engineered NK cells generate extracellular vesicles that inherit CAR molecules together with membrane-associated receptors and cytotoxic proteins from their parental cells. Consequently, CAR-NK cell-derived EVs integrate antigen-specific recognition with the intrinsic cytotoxic machinery of NK cells, providing a versatile platform for targeted cell-free immunotherapy (4, 46–49).

Importantly, the extent to which parental CAR expression translates into functional CAR incorporation on individual EVs remains insufficiently quantified. Future studies should determine the fraction of CAR-positive EVs, CAR density and orientation, antigen-binding activity, and differential uptake by antigen-positive versus antigen-negative cells, together with *in vivo* tumor-to-organ distribution.

CAR-NK cell-derived EVs can further encapsulate therapeutic agents such as cytokines, siRNAs, or immune checkpoint inhibitors to simultaneously target tumor growth and modulate the immune microenvironment (20, 42, 49). Combination with immune checkpoint blockade may provide complementary mechanisms of action, as CAR-NK cell-derived EVs can deliver cytotoxic and immunomodulatory cargo while checkpoint inhibition may restore the activity of endogenous T and NK cells suppressed within the tumor microenvironment. Such combinations could therefore enhance tumor-cell killing while relieving adaptive immune resistance; however, evidence specifically evaluating CAR-NK cell-derived EVs in combination with immune checkpoint blockade remains limited and requires direct validation in appropriate models (20, 42, 43, 49).

For nucleic-acid delivery, lipid nanoparticles (LNPs) provide an important benchmark because they offer mature formulation and manufacturing platforms, efficient nucleic-acid encapsulation, and demonstrated clinical translation (50, 51). CAR-NK cell-derived EVs may offer advantages related to their biological membrane composition and intrinsic cell-derived targeting properties, but they face greater challenges in compositional heterogeneity, loading reproducibility, large-scale manufacturing, and regulatory standardization (50). Thus, CAR-NK cell-derived EVs should currently be considered a complementary rather than intrinsically superior platform to LNPs for nucleic-acid delivery.

In addition to genetic engineering, surface functionalization of EV enhances their targeting capacity through ligand–antibody conjugation. Ligands that target receptors highly expressed on tumors or tumor vasculature increase EVs homing efficiency, overcoming barriers posed by tumor heterogeneity and dense extracellular matrices (52, 53).

These modifications reduce off-target effects, improving safety while maintaining precise delivery (42, 48). CAR-NK cell-derived EVs may offer practical advantages over some conventional cell-based approaches by avoiding several risks associated with administration of viable immune cells, including uncontrolled cellular proliferation and cell-mediated toxicities; however, the safety profile of EV products must be established independently through dedicated preclinical studies (20, 27, 46).

Moreover, like other extracellular vesicles, the nanoscale size of CAR-NK cell-derived EVs facilitates penetration into solid tumors through dense extracellular matrices. Beyond improved tissue penetration, these vesicles may also mitigate the functional consequences of hypoxia within the tumor microenvironment by delivering cytotoxic proteins, immunoregulatory molecules, and regulatory RNAs that preserve immune-cell activity, attenuate hypoxia-driven immunosuppressive signaling, and enhance antitumor immune responses. Although these mechanisms remain incompletely understood, accumulating evidence suggests that engineered EVs can partially counteract hypoxia-associated immune dysfunction rather than merely diffuse through hypoxic regions (20, 42, 43, 54).

These features suggest that CAR-NK cell-derived EVs could be considered as a potential cell-free option in settings where viable cell therapy is difficult to administer (34, 47). Owing to their modular design, CAR-NK cell-derived EVs can support multifunctional applications such as delivering synergistic payloads, for example; siRNAs combined with anti-PD-1 antibodies, or integrate imaging agents for theranostic purposes (42, 55). They may also enhance chemotherapy/radiotherapy through immune activation (55). Moreover, strategies such as using PEGylation or biomimetic membrane coatings (e.g., red blood cell-derived membranes) can prolong the EV circulation time and tumor accumulation and enhance their therapeutic potential, although their effects are context-dependent and require empirical validation (49, 54).

A major challenge in cancer treatment is the immunosuppressive nature of the TME, which enables tumors to evade immune surveillance and resist therapy. CAR-NK cell-derived EVs provide a novel strategy to counteract these challenges by modulating the TME to favor anti-tumor immunity. These EVs can be engineered to carry immunostimulatory molecules, such as cytokines (e.g., IL-15 or IFN-γ) and chemokines, which attract and activate effector immune cells such as cytotoxic T lymphocytes and dendritic cells (DC). This activity not only strengthens the immune response but also disrupts the immune-suppressive network maintained by the tumor (34, 36, 52).

In addition to modulating the TME, CAR-NK cell-derived EVs possess inherent cytotoxic capabilities that directly target and kill tumor cells. These EVs are enriched with cytolytic molecules such as perforins and granzymes, which can induce apoptosis in cancer cells upon delivery. Furthermore, they carry natural ligands that activate death receptors on tumor cells, triggering cell death pathways and promoting tumor regression. This dual mechanism - modifying the TME and exerting direct cytotoxic effects - renders CAR-NK cell-derived EVs highly effective in overcoming immune escape mechanisms employed by cancer cells (12, 19, 47).

Another critical advantage is their ability to target tumor-associated stromal cells that support tumor growth and metastasis. By disrupting supportive niches within the TME, such as the premetastatic niches identified in recent studies, CAR-NK cell-derived EVs can inhibit tumor progression and metastasis (20, 49, 56).

CAR-NK cell-derived EVs may ultimately provide an off-the-shelf therapeutic format; however, scalable production currently remains a major translational challenge requiring efficient expansion of CAR-NK cells, controlled EV recovery and purification, standardized potency testing, and reproducible GMP-compatible manufacturing (34). Unlike CAR-T, which require complex manufacturing, EVs maintain stability during cryopreservation and distribution, but these advantages do not by themselves establish superior scalability or manufacturing feasibility (57). These issues are particularly important in resource-limited settings where access to advanced cellular therapies remains challenging (58).

Therefore, CAR-NK cell-derived EVs represent a potential platform linking cellular immunotherapy and nanomedicine, with the possibility of combining targeted recognition, cytotoxic activity, and TME modulation. However, their relative safety, efficacy, scalability, and clinical utility remain to be established in rigorous product-specific studies (**Figure 3**).

**Figure 3:**
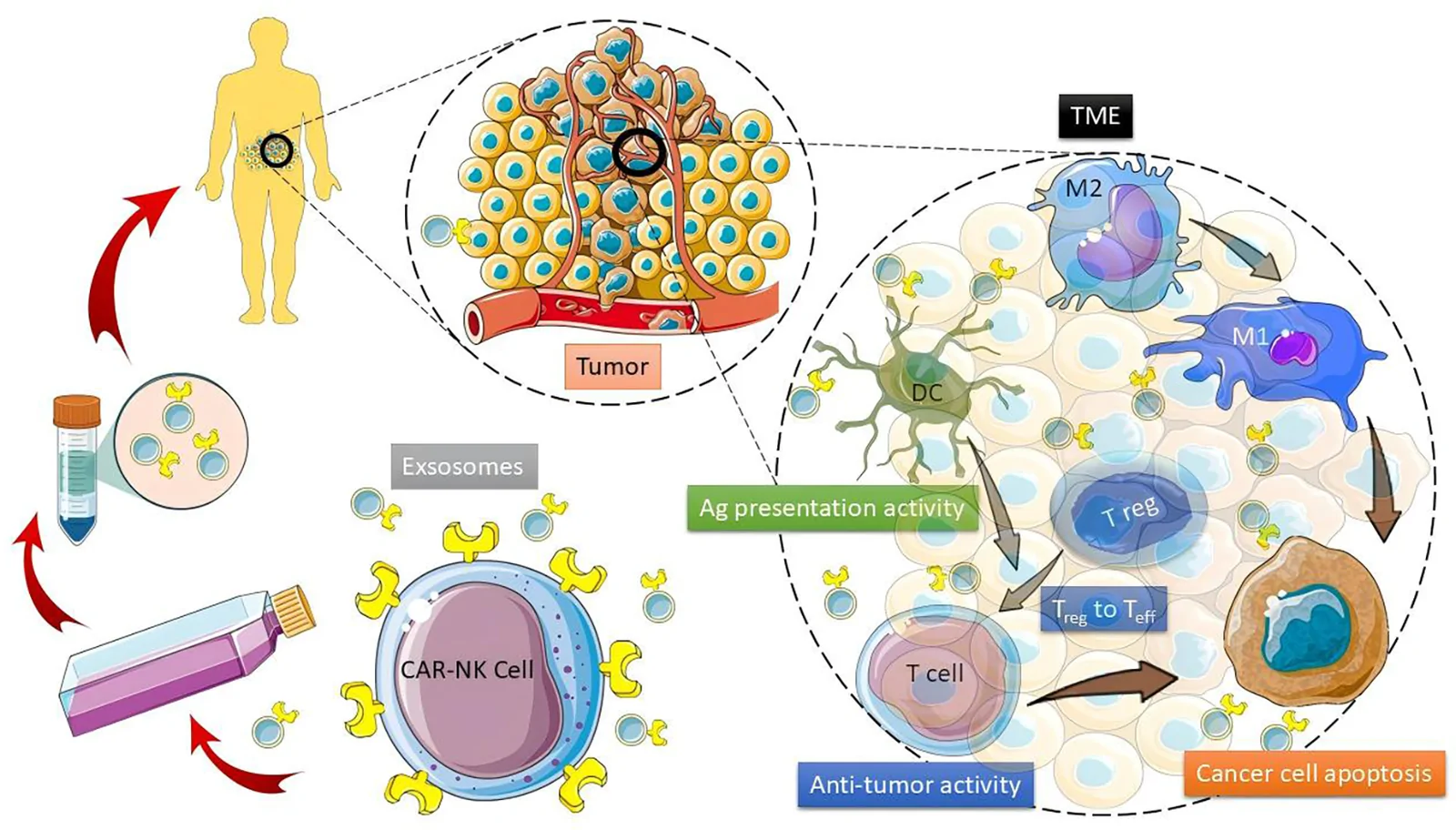
This figure illustrates the therapeutic advantages of CAR-NK cell-derived EVs for targeting tumors within the TME. The diagram begins with collection of CAR-NK cell-derived EVs from culture medium. Following administration, these EVs may reach tumor tissue and interact with tumor and immune cells. Proposed effects include enhanced antigen-presenting activity of DCs, modulation of regulatory T-cell and effector-cell responses, and polarization of tumor-associated macrophages toward antitumor phenotypes. These mechanisms may contribute to cancer-cell apoptosis and TME remodeling, but their magnitude and relevance require experimental validation. TME, Tumor Microenvironment; DC, Dendritic Cell; T reg, Regulatory T cells; T effs, Effector T Cell.

## 3 Current status of research and clinical translation

Building upon the unique biological properties discussed above, CAR-NK-derived extracellular vesicles have emerged as promising cell-free immunotherapeutic agents for both hematological and solid malignancies. By combining antigen-specific targeting with the intrinsic cytotoxic machinery inherited from their parental NK cells, these vesicles offer a versatile platform capable of direct tumor killing while simultaneously modulating the tumor immune microenvironment (18–20, 38). This section provides an overview of the current preclinical and clinical status studies focused on these innovative therapies in cancer treatment.

### 3.1 Preclinical findings

#### 3.1.1 *In-vitro* studies

Preclinical investigations have highlighted the efficacy of CAR-NK cell-derived EVs *in vitro*, demonstrating their potential to selectively target and destroy tumor cells. These EVs are capable of delivering cytotoxic molecules, including perforins and granzymes, directly to tumor cells, inducing apoptosis and reducing tumor cell viability (4, 59). One notable strategy involves engineering CAR-NK cell-derived EVs to deliver cytokines such as interleukin-15 (IL-15). IL-15 has been shown to not only enhance the cytolytic activity of NKs but also recruit and activate other immune cells, thereby amplifying the immune response against cancer cells (60).

NK-cell-derived EVs have also been explored as vehicles for chemotherapeutic drugs. Paclitaxel-loaded NK-cell EVs enhanced delivery of paclitaxel to tumor cells in preclinical models (61). Similarly, NK-cell-derived EVs loaded with cisplatin enhanced antitumor activity in an ovarian cancer model (62). These findings should be regarded as indirect evidence supporting the broader NK-EV platform rather than direct evidence for CAR-NK cell-derived EVs.

Light-Activatable Silencing NK cell-Derived Exosome (LASNEO) is an engineered NK-cell-derived EV platform based on light-triggered therapeutic activation. Preclinical studies reported immune-cell recruitment, silencing of tumor-promoting genes, and enhanced antitumor activity following light activation (63). Because the reported LASNEO study used NK-cell-derived EVs rather than CAR-NK cell-derived EVs, it provides indirect engineering and mechanistic context for the CAR-NK-EV field. This approach allows for precise control over therapeutic delivery, reducing off-target effects and can be used in CAR-NK cell-derived EVs (**Figure 1**).

These findings underscore the versatility of CAR-NK cell-derived EVs as platforms for both immunomodulation and drug delivery in cancer therapy (**Table 1**). However, as direct studies of CAR-NK cell-derived EVs remain limited, evidence from conventional NK-derived EVs and related engineered or biomimetic systems is included where it provides mechanistic or technological context; these studies should not be interpreted as direct evidence of CAR-NK-EV efficacy.

**Table 1:** Preclinical evidence supporting NK-cell-derived and CAR-NK-derived extracellular vesicle therapeutics in cancer.

Study	Cancer type	Therapeutic approach	Key findings	Evidence category	References
Exosomes Derived from Natural Killer Cells Exert Therapeutic Effect in Melanoma	Melanoma	NK Cell-Derived Exosomes	NK cell-derived exosomes exhibited significant antitumor effects by promoting immune activation and tumor cell targeting.	Indirect: Natural NK-EV evidence	(4)
On-Chip Biogenesis of Circulating NK Cell-Derived Exosomes in Non-Small Cell Lung Cancer Exhibits Antitumoral Activity	Non-Small Cell Lung Cancer	Engineered NK Cell-Derived Exosomes	Engineered exosomes from NK cells showed potent antitumoral effects *in vivo*, improving the overall tumor suppression and immune modulation.	Indirect: Engineered NK-EV evidence	(68)
Engineering CAR-NK Cell Derived Exosome Disguised Nano-Bombs for Enhanced HER2 Positive Breast Cancer Brain Metastasis Therapy	HER2+ Breast Cancer	CAR-NK Cell Exosomes Disguised as Nano-Bombs	CAR-NK-derived exosomes delivered therapeutics specifically to brain metastasis, significantly reducing tumor burden and metastasis.	Direct CAR-NK-EV evidence	(49)
Natural Killer Cell-Derived Exosome-Based Cancer Therapy	Various Cancer Types	Exosome-Based Therapeutics	Natural killer cell-derived exosomes demonstrated the ability to reprogram the TME and facilitate targeted drug delivery.	Review/contextual evidence	(12)
Enhanced Antitumor Activity in Melanoma Using IL-15-Primed NK Cell-Derived Exosomes	Melanoma	IL-15-Primed NK Cell-Derived Exosomes	IL-15 priming enhanced the antitumor potency of exosomes, leading to improved immune activation and tumor suppression in melanoma models.	Indirect: Engineered NK-EV evidence	(60)
Paclitaxel-Loaded NK Cell-Derived Exosomes for Breast Cancer Therapy	Breast Cancer	Paclitaxel-Loaded NK Cell-Derived Exosomes	These exosomes enhanced the delivery of paclitaxel to tumor cells, increasing therapeutic efficacy while reducing systemic toxicity.	Indirect: Engineered NK-EV evidence	(61)
Cytokine-Enhanced Cytolytic Activity of Exosomes from NK Cells	Various Cancers	Cytokine-Enhanced NK Cell Exosomes	Exosomes preloaded with cytokines exhibited enhanced cytolytic activity, improving immune system activation and tumor cell apoptosis.	Indirect: Engineered NK-EV evidence	(59)
Natural Killer-Derived Exosomal miR-186 Inhibits Neuroblastoma Growth	Neuroblastoma	NK Cell-Derived Exosomes Loaded with miR-186	miR-186-loaded exosomes inhibited tumor growth and immune escape mechanisms, reprogramming the TME.	Indirect: Engineered NK-EV evidence	(65)
Mass-Spectrometric Proteome Analysis of NK-92 Cell-Derived Microvesicles	Various Cancers	Proteome Analysis of NK-92 Cell Microvesicles	Identified proteins responsible for immune activation, providing insights into the cytolytic mechanisms of NK-derived vesicles.	Mechanistic/characterization evidence	(69)
Broad and Potent Anti-Tumor Activity of NK-3.3 Cell-Derived Extracellular Vesicles	Various Solid Tumors	NK-3.3 Cell-Derived Extracellular Vesicles	NK-3.3 cell-derived vesicles showed broad anti-tumor effects, promoting immune responses and tumor suppression in multiple models.	Indirect: Natural NK-EV evidence	(70)
Natural Killer Cell Membrane-Infused Biomimetic Liposomes for Tumor Therapy	Various Cancers	NK Cell Membrane-Infused Biomimetic Liposomes	Liposome-based carriers combined the specificity of NK cell membranes with enhanced delivery of therapeutic agents to tumors.	Indirect: Biomimetic NK-derived platform	(71)
Light-Activatable Silencing NK cell-Derived Exosome (LASNEO) for Synergetic Tumor Eradication	Various Cancers	Light-Activatable NK Cell-Derived Exosomes	LASNEO exosomes recruited immune cells and silenced tumor-promoting genes, achieving synergistic tumor suppression with light activation.	Indirect: Engineered NK-EV evidence	(63)
Therapeutic Silencing of BCL-2 Using NK Cell-Derived Exosomes in Breast Cancer	Breast Cancer	NK Cell-Derived Exosomes Silencing BCL-2	Exosomes silencing BCL-2 effectively inhibited tumor growth and induced apoptosis in breast cancer models.	Indirect: Engineered NK-EV evidence	(72)
NK Cell-Derived Exosomes Enhance Anti-Tumor Effects in Ovarian Cancer	Ovarian Cancer	NK Cell-Derived Exosomes Loaded with Cisplatin	Exosomes loaded with cisplatin enhanced anti-tumor efficacy by delivering the drug directly to tumor cells and reactivating NK cell functions.	Indirect: Engineered NK-EV evidence	(62)

#### 3.1.2 *In-vivo* studies

*In vivo* studies provide direct evidence that some CAR-NK cell-derived EV preparations can have antitumor activity in animal models. For example, in HER2+ breast cancer models, CAR-NK cell-derived EVs achieved 70% tumor growth inhibition and extended survival by 40% compared with controls. Similarly, EVs targeting BCL-2 silence antiapoptotic genes, inducing apoptosis and halting tumor progression (49). Nevertheless, these studies provide indirect evidence and should not be interpreted as direct validation of CAR-NK cell-derived EVs (64).

Another key finding *in vivo* is the ability of CAR-NK cell-derived EVs to modulate the TME. These EVs can suppress immunosuppressive factors within the TME while enhancing the infiltration and activation of effector immune cells, such as cytotoxic T lymphocytes and natural killer cells. This dual activity contributes to a more sustained and comprehensive antitumor response (20). Studies using melanoma models have demonstrated that CAR-NK cell-derived EVs carrying tumor-suppressive microRNAs, such as miR-186, can effectively inhibit tumor progression and immune escape mechanisms (components of the *TGF-β* signaling pathway) (20, 65).

CAR-NK cell-derived EVs have also demonstrated immune-enhancing effects in neuroblastoma models by increasing the potency of cytokine-activated NK cells (66). Similarly, NK-cell-derived EVs have been reported to influence tumor-associated macrophages and dendritic cells within the TME (28, 67). These findings provide mechanistic context, but should be distinguished from direct evidence obtained with CAR-NK cell-derived EVs.

This reprogramming not only diminishes the suppressive effects of these cells but also converts them into immune-supportive phenotypes, thereby augmenting the overall antitumor immune response (20). In models of solid tumors, this combination of direct cytotoxicity and TME reprogramming has demonstrated favorable results, paving the way for novel multipronged cancer therapies.

These preclinical findings collectively underscore the versatility and therapeutic potential of CAR-NK cell-derived EVs, not only as standalone treatments but also as components of combination therapies. Further optimization and translation of these strategies into clinical settings are critical next steps to maximize their impact in oncology.

### 3.2 Clinical translation readiness

Clinical experience with CAR-NK cells provides important context for the translational potential of the parental cellular platform, but it should not be interpreted as clinical evidence for CAR-NK cell-derived EVs. In a pivotal phase 1/2 study of cord-blood-derived CD19-directed CAR-NK cells, 8 of 11 treated patients (73%) achieved an objective response and 7 of 11 (64%) achieved a complete response, with responses observed without major treatment-related toxicities such as severe cytokine release syndrome, neurotoxicity, or graft-versus-host disease (45). Other early CAR-NK studies have reported encouraging activity in hematological malignancies, whereas clinical experience in solid tumors remains limited (21, 46).

In contrast, CAR-NK cell-derived EVs have not yet entered clinical trials, and no clinical efficacy or safety data are currently available for this specific cell-free product. In the United States, the FDA states that exosome products intended to treat diseases or conditions in humans are regulated as drugs and biological products and require FDA review; no exosome product is currently FDA-approved (73). Accordingly, clinical development would require an investigational new drug (IND)-enabling package addressing product identity, purity, potency, manufacturing consistency, and chemistry, manufacturing, and controls (CMC), together with appropriate pharmacology, toxicology, biodistribution, pharmacokinetic/pharmacodynamic (PK/PD), and clinical-study information. FDA guidance for CAR T-cell products notes that relevant CMC, pharmacology/toxicology, and clinical-development principles may also inform development of genetically modified lymphocyte products such as CAR-NK cells, while product-specific considerations should be discussed with FDA (74). The CMC framework for INDs further emphasizes control of product safety, identity, quality, purity, and potency (75). For CAR-NK cell-derived EVs, these requirements will need to be adapted to the final cell-free product and its manufacturing process, and its critical quality attributes.

## 4 CAR-NK cell-derived EVs: limitations and translational challenges

CAR-NK cell-derived EV therapy faces several challenges, including large-scale production, EV heterogeneity, limited understanding of biodistribution and pharmacokinetics, and the absence of clinically validated dose and potency standards (3, 37, 76, 77). These limitations are addressed in detail below.

### 4.1 Challenges in production and scalability

The therapeutic potential of CAR-NK cell-derived EVs is overshadowed by the significant challenges associated with their production and scalability. Current methods for isolating and purifying EVs from cell culture supernatants are labor intensive, time consuming, and expensive. Conventional ultracentrifugation requires extensive processing and may result in variable recovery, potentially affecting product consistency (29, 36). Producing therapeutically meaningful quantities also requires expansion of the parental CAR-NK population, conditioned-medium collection, concentration, purification, characterization, sterile processing, and potency testing. Thus, claims that CAR-NK cell-derived EVs are inherently easier or cheaper to manufacture than cellular therapies remain premature until quantitative comparisons of cell numbers, culture volume, EV yield, recovery, processing time, cost, and batch reproducibility are available.

Innovations in production technology, such as bioreactor systems designed for large-scale cell culture, may help address these scalability issues. Additionally, advancements in EVs isolation methods, including size-exclusion chromatography and microfluidic technologies, could improve the efficiency and reproducibility of EVs production while reducing costs. However, these approaches require validation for CAR-NK cell-derived EV products (32, 42).

EV-mimetic nanovesicles may offer greater production control and scalability, but they should be considered distinct engineered platforms rather than interchangeable with naturally derived EVs (33, 42). In general, EVs isolation and purification methods need to be standardized to minimize batch-to-batch variation and achieve the commercial viability of CAR-NK cell-derived EVs therapies to meet the growing demand for consistent, high-quality therapeutic products (12).

### 4.2 Exosome heterogeneity

Another limitation in CAR-NK cell-derived EVs therapy lies in the inherent heterogeneity of EVs preparations. EVs exhibit significant variability in terms of size, surface markers, and cargo composition, even when derived from the same cell population. This heterogeneity can affect targeting efficiency, payload delivery, potency, and reproducibility (42, 78).

Variability in cargo content, such as protein, lipid, and RNA contents, can lead to inconsistencies in the therapeutic effects observed in preclinical and clinical studies and the efficient loading of therapeutic agents into CAR-NK cell-derived EVs remains challenging. Emerging techniques, including electroporation, sonication, and lipid fusion, demonstrate improved loading efficiencies without compromising vesicle stability (20, 79). For clinical translation, cargo-engineering studies should also report standardized parameters including encapsulation efficiency, cargo-to-particle or cargo-to-EV-protein ratios, recovery yield, release kinetics, cargo stability, and changes in vesicle size and surface charge after loading. Furthermore, heterogeneity in surface markers may reduce the ability of EVs to selectively bind to and penetrate tumor cells (33). Addressing this issue requires standardized isolation and purification protocols, as well as the development of manufacturing processes that ensure consistency in EVs quality. Engineering EVs with uniform surface modifications, synthetic scaffolds or the incorporation of targeting peptides or antibodies, may hold promise in reducing heterogeneity, enhancing the reproducibility, increasing tumor specificity and minimizing off-target effects ^12,46,49,57.^

### 4.3 Biodistribution and clearance

Despite their ability to circulate in the bloodstream and penetrate tumor tissues, the biodistribution and pharmacokinetics of EVs *in vivo* remain poorly understood. Factors such as particle size distribution, surface charge, membrane composition, surface proteins, aggregation state, and adsorption of serum proteins forming a protein corona can influence their circulation time, recognition by the mononuclear phagocyte system, tissue penetration, and accumulation in organs such as the liver, spleen, and lung. Interpretation of EV biodistribution is further influenced by the administration route, dose, labeling strategy, and surface engineering, while the lack of standardized protocols limits comparison across studies and optimization for clinical applications (36, 76). Evidence from NK-cell-derived EV studies indicates that liver and spleen are important clearance sites and that tumor-associated distribution can occur after systemic administration. For example, a review of NK-derived EV studies reported tumor-associated signal as early as 12 hours after intravenous administration in a glioblastoma model, with peak signal at approximately 48 hours, while IL-15-primed NK-EVs were reported to have a circulation half-life of up to 24 hours in mice (60). These observations provide useful context but cannot be directly extrapolated to CAR-NK cell-derived EVs. Moreover, EVs while smaller than whole cells, can still accumulate in various organs, leading to off-target effects that may reduce the efficacy of therapy or result in unintended consequences (57). The interaction of CAR-NK-EVs with the immune system and the TME adds another layer of complexity. For example, EVs may be cleared by macrophages or other components of the mononuclear phagocyte system, reducing their therapeutic efficacy. Strategies to increase the stability and circulation time of EVs, such as PEGylation or surface engineering with immune-evasive molecules, are being explored to address these challenges (42, 55, 80). Moreover, CAR-NK cell-derived EVs loaded with immune checkpoint inhibitors or proinflammatory cytokines offer a novel approach to reprogramming the TME (12, 57). These vesicles can promote immune cell infiltration, disrupt tumor-supportive stromal networks, and enhance the recognition of cancer cells by the immune system (38, 79).

Future preclinical development should also incorporate repeat-dose toxicology, immunogenicity assessments, potential accumulation in clearance organs, and rechallenge studies to determine the durability of antitumor protection. Future research should focus on in-depth characterization of the pharmacokinetics, bio-distribution, and interaction mechanisms of CAR-NK cell-derived EVs *in vivo*. This understanding will be critical for optimizing their design and delivery, thereby improving their therapeutic potential and accelerating their translation into clinical practice.

### 4.4 Safety considerations

The cell-free nature of CAR-NK cell-derived EVs may reduce risks associated with viable-cell administration, but it should not be equated with an established favorable safety profile. No quantitative clinical safety incidence data are currently available for CAR-NK cell-derived EVs because these products have not entered clinical trials. EVs are biologically active particles and may contain or acquire molecules that influence angiogenesis, stromal remodeling, or metastatic niches; therefore, source-cell state, cargo composition, and off-target effects require systematic assessment (12, 57). In addition, hepatosplenic uptake by macrophages and other components of the mononuclear phagocyte system may contribute to off-target accumulation and altered exposure (57, 76). Engineered EVs may also carry cytokines or other immunomodulatory cargo capable of contributing to cytokine release or other immune-mediated adverse effects. Repeat-dose studies should assess changes in biodistribution, anti-EV antibody responses, complement or other innate immune activation, cumulative toxicity, and loss or alteration of pharmacodynamic activity (12, 76). These considerations are particularly important because evidence supporting safety remains predominantly preclinical.

### 4.5 Pharmacokinetic and pharmacodynamic considerations

Clinical translation will require quantitative PK/PD characterization of CAR-NK cell-derived EVs rather than extrapolation from cell-based CAR-NK therapy. Available NK-EV evidence provides useful preliminary context: systemic EV administration is associated with rapid clearance and substantial uptake by phagocytic cells, with liver and spleen representing important distribution and clearance sites. In mouse models, NK-derived EVs have shown tumor-associated distribution within hours after intravenous administration, and IL-15-primed NK-EVs have been reported to exhibit a circulation half-life of up to 24 hours (60). These findings are not equivalent to CAR-NK-EV PK data and should not be used to define a clinical dose. PK studies should report, where feasible, Cmax, area under the curve, apparent half-life, clearance, tissue accumulation, and tumor-to-organ exposure ratios. PD studies should assess target engagement, cellular uptake, cytotoxic activity, immune-cell modulation, and relevant pharmacodynamic biomarkers. No standardized dosing regimen currently exists for CAR-NK cell-derived EVs; future studies should therefore define dose on a reproducible basis (e.g., particle number and/or EV protein), establish dose-response and exposure-response relationships, and evaluate administration route, dosing interval, and repeat dosing. PEGylation and related surface-engineering strategies may prolong circulation and alter biodistribution, but they can also affect cellular uptake, CAR accessibility, and biological activity; therefore, they require empirical optimization and should be evaluated using both systemic exposure and tumor-to-clearance-organ distribution endpoints (76, 80).

## 5 Future research directions

Although CAR-NK-derived extracellular vesicles have demonstrated remarkable therapeutic potential in preclinical studies, several scientific and technological challenges must be overcome before they can become clinically applicable. Future research should therefore focus not only on improving anti-tumor efficacy but also on robust manufacturing processes, standardized characterization, clinically relevant quality-control criteria, quantitative PK/PD, dose selection, and product-specific safety assessment. Progress in these areas will be essential for translating CAR-NK cell-derived EVs from experimental platforms into reproducible therapeutic products.

One important direction involves the continued optimization of CAR engineering and extracellular vesicle design. Future studies should explore NK-specific CAR architectures, strategies to enhance extracellular vesicle secretion, and methods for improving cargo loading efficiency while preserving vesicle integrity. Surface functionalization with tumor-targeting ligands, antibodies, or biomimetic membranes may further increase tumor specificity, prolong circulation time, and improve biodistribution. Likewise, combining CAR-NK cell-derived EVs with therapeutic payloads such as cytokines, immune checkpoint inhibitors, nucleic acids, or conventional anticancer drugs may generate multifunctional nanotherapeutics capable of simultaneously targeting multiple mechanisms of tumor progression (8, 12, 42, 53).

Beyond engineering advances, a deeper understanding of interactions between CAR-NK cell-derived EVs and the tumor microenvironment will be crucial. Although accumulating evidence suggests that these vesicles can modulate immune-cell function, remodel immunosuppressive niches, and enhance antitumor immunity, the molecular mechanisms governing these effects remain incompletely understood. Future investigations should therefore clarify how engineered EVs influence macrophages, dendritic cells, T lymphocytes, stromal cells, and extracellular matrix remodeling across different tumor types, as well as identify biomarkers that predict therapeutic responsiveness (20, 28, 43, 81).

Equally important are advances in manufacturing and computational technologies. The development of scalable bioreactor-based production systems, standardized isolation procedures, and reliable potency assays will be indispensable for clinical translation. At the same time, artificial intelligence and machine-learning approaches may accelerate several stages of CAR-NK cell-derived EV development by facilitating CAR design, optimizing cargo selection, predicting tissue distribution, identifying novel target antigens, and supporting individualized treatment strategies based on tumor molecular profiles. The integration of computational modeling with experimental validation may therefore substantially accelerate the development of next-generation engineered EV therapeutics (32, 42, 47, 80).

Finally, successful clinical translation will require carefully designed early-phase clinical trials evaluating safety, biodistribution, pharmacokinetics, optimal dosing schedules, and long-term efficacy across both hematological and solid malignancies. Given their cell-free nature, engineering flexibility, and potential for off-the-shelf development, CAR-NK-derived extracellular vesicles represent a promising next generation of precision immunotherapeutics, although their safety profile requires dedicated preclinical and clinical validation. Continued multidisciplinary collaboration among immunologists, bioengineers, nanomedicine researchers, computational scientists, and clinicians will be essential to realize their full clinical potential.

## 6 Conclusion

CAR-NK-derived extracellular vesicles represent an emerging generation of cell-free immunotherapeutics that combine the antigen specificity of CAR engineering with the intrinsic cytotoxic and immunomodulatory properties of natural killer cells. Their nanoscale architecture, engineering flexibility, and cell-free format position them as attractive candidates for overcoming several limitations associated with conventional adoptive cellular therapies, particularly in the context of solid tumors.

Although substantial progress has been achieved in preclinical models, important challenges remain before clinical implementation becomes feasible. These include standardizing extracellular vesicle manufacturing and characterization, improving cargo loading and targeting efficiency, defining pharmacokinetic and biodistribution profiles, and establishing robust potency assays for regulatory approval. Addressing these issues will require coordinated efforts across immunology, extracellular vesicle biology, bioengineering, nanomedicine, and translational oncology.

Overall, CAR-NK-derived extracellular vesicles are unlikely to replace cellular immunotherapy but instead are expected to complement existing therapeutic strategies. Continued advances in engineering technologies, scalable manufacturing, and mechanistic understanding will determine whether these engineered vesicles can fulfill their promise as clinically applicable precision immunotherapeutics that bridge cellular immunotherapy and nanomedicine.
